# Book Reviews

**Published:** 1826-09

**Authors:** 


### CRITICAL ANALYSES.

Quæ laudanda forent, et quæ culpanda, vicissim

Illa, prius, cretâ; mox hæc, carbone, *notamus*.—PERSIUS.

*Observations on the Physiological Principle of Sympathy, chiefly in reference to the peculiar Doctrines of* Mr. Charles Bell. By W. P. Alison, m.d. f.r.s.e.; Secretary to the Society, and Joint-Professor of the Theory of Medicine in the University of Edinburgh. (*Transactions of the Medico-Chirurgical Society of Edinburgh,* vol. ii.)

Mr. Charles Bell, in his paper on the Nervous Circle,* has proved, by means of anatomy, a physiological truth of great importance,—namely, that a sensation, conveyed to the mind, precedes the action of a muscle. We must remark, however, that, in the paper alluded to, this principle has been followed out only so far as to show the connexion of the nerves of sensation with the muscles of voluntary action. Yet there is no reason assigned for confining the connexion to the voluntary muscles: on the contrary, some of the illustrations prove that the same principle applies equally to those which are involuntary. The nerve to which our attention is most frequently directed, is the portio dura, the motor nerve of the muscles of the face; and we know that this, at certain times, is under the control of the will, but most frequently is independent of it. We shall, by and by, have occasion to dwell upon the principle which we have now stated, and we shall then show that it is applicable both to voluntary and involuntary actions.

About the same time that Mr. Bell's paper was published, Dr. Alison's essay also appeared. The chief object of this is to refute Mr. Bell's doctrines; and we proceed to show the grounds on which Dr. Alison founds his objections to the classification of the nerves as proposed by that author.

Mr. Bell's last paper has for its object to prove that there is a nervous connexion between muscular actions and sensations in the brain: and *he* illustrates the subject by anatomy, referring to the motor nerves and the nerves of sensation. Dr. Alison's paper has for its object to prove the principle of "all sympathetic actions being dependent on mental sensations:" *his* reasoning is founded on metaphysical proofs. Thus there is an essential difference in the mode by which these two gentlemen arrive at nearly the same general prin-

* Philosophical Transactions, 1826.

ciple: the former grounds his reasoning on anatomy; the latter rejects anatomy altogether.

Dr. Alison has professed to make his observations on this subject, only somewhat fuller, illustrations of the principle laid down by Dr. Whytt, in his "Essay on the Sympathy of the Nerves." It is, perhaps, known to most of our readers, that the theory of Dr. Whytt has been long regarded as exceedingly vague and uncertain, because its consistency with the anatomy of the nervous system could not be proved:* yet this very circumstance appears to enhance its value in the opinion of our author. "The researches of Dr. Whytt and Dr. Munro on this subject were, as I think, quite successful in establishing two points in regard to such phenomena: first, that they cannot be explained by the connexion of the nerves of the sympathising parts; and, secondly, that they do not indicate any necessary consent or sympathy between the individual parts of the body, but are in general simply the effects of certain mental sensations." (P. 170.) In these sentiments we find an explanation of the reason why Dr. Alison refuses to adopt Mr. Bell's views: he has perceived that the class of respiratory nerves is made to account for several sympathetic actions, and, being a disciple of Drs. Whytt and Munro, and convinced by them that the principle of sympathetic actions cannot be explained by the connexions of nerves, he has concluded that Mr. Bell's views are incompatible with their doctrine; and therefore that the functions of the nerves, as explained by him, must be unfounded.

As this is a very curious and important subject, we shall endeavour to show that the connexions of the nerves do assist us in understanding the principle of sympathy; and that the new classification of the nerves affords us the explanation which Dr. Alison thinks impossible.

Although this gentleman has stated his conviction that sympathetic actions depend upon sensation, yet he has omitted to consider the nature and peculiarities of different sensations. Having arrived at the conclusion, that there is a "previous sensation in the mind," he conceives that he has attained the whole physiological truth concerning the principle of sympathy; and he has, perhaps, advanced so far as

* If we refer to the numerous papers written during Haller's time on the same subject, we shall find that this principle forms the foundation of many rival theories, as in the following:—By Joannes Astruc, "An Sympathia Partium a certa Nervorum Positura in interno Sensorio?" 1736; Albrecht Thaer, "De Actione Systematis Nervosi in Febribus," Goettingæ, 1774; Christ. Gul. Henr. de Marees, "De Animi Perturbationum in Corpus Potentia," Goettingæ, 1775; Ernestus Platnerus, "De Causis Consensus Nervorum Physiologicis," Lipsiæ, 1790; and many others.

metaphysical research allowed. But it is to be considered —1st, that sensibilities are infinitely varied over the whole body; 2ndly, that each kind of sensibility is attended with its appropriate sympathetic effects; and 3dly, that the nerves which convey sensation to the sensorium form a distinct class.

1. The nerves of sensation are distributed, as we well know, over the whole body: their most striking peculiarity is, that the sensibilities, which they convey to the sensorium, differ in almost every part of the body. By a wise provision of nature, each organ is endowed with that kind of sensibility which is best suited for its protection, and its offices in the economy. Thus, the common integument is not endowed with a sensibility which resembles that of the surface of the eye; the heart does not possess the same sensibility as the skin, yet it is most acutely sensible to its proper stimulus,—viz. the distention by blood; ligaments, tendons, and cartilages, possess sensibility of a certain kind, yet not such as to bestow pain during the natural motions of the body; but, if a joint be stretched beyond its proper degree, its sensibility is immediately called into action, as any one who has sprained his ankle must know full well.

These examples are sufficient to prove that sensations are different in the various structures of the body. They are implanted for the purpose of guarding the individual parts; and it is in conformity with this principle that each structure is endowed with an appropriate defence against injury.

2. Sensations likewise vary throughout the organs of the body, to produce certain combinations of the muscles.

If we consider the system of the animal body, we shall see many instances of peculiar sensibilities rousing certain muscles into activity. Irritation of the larynx, producing cough, is not a more curious example of a peculiar sensation stimulating an infinite number of large and small muscles, to combine for one purpose, than irritation produced by the pressure of the urine upon a particular spot of the bladder, in causing some muscles to relax, and others to contract, for the expulsion of the urine. While considering the muscles of the perineum in the male, we find another example, equally illustrative: for, when a different kind of sensation forms the excitement, a different combination of the muscles is produced. The arrangement in the action of the muscles for expelling the fæces, forms also another illustration of this subject. We might multiply our instances by referring to the intestines, or to the heart, which contract on their contents, each acknowledging the influence of its proper stimulus. No example is more wonderful than that of the sensibility of the uterus, which, having remained inactive during gestation, at length contracts upon the fœtus, when the proper period for its expulsion has arrived.

These examples show that certain sensibilities are implanted in various parts of the body, which, being excited, cause the actions of appropriate muscles. Each of these sensibilities is bestowed for useful purposes in the economy; and it is necessary that we should have a perfect acquaintance with the functions of a part, to understand the character of its proper sensibility.

3. Let us now consider the class of nerves which bestow sensation.

The anatomical fact, in relation to the variety of the sensibilities in the body, is this :—The nerves of sensation, which are those arising from the posterior column of the spinal marrow and the fifth pair, are endowed with sensibilities very differently modified. This may be best illustrated by considering the fifth pair. It is now ascertained that this nerve bestows, first, the degree of sensation which is peculiar to the skin ; secondly, that acute sensibility which is possessed by the surface of the eye; thirdly, the sensation peculiar to the internal membrane of the nose; fourthly, that of the sides and tip of the tongue; and, fifthly, that of the muscles. Instances have occurred to prove that any of these sensibilities may be separately lost by affections of the fifth pair.

But, if we inquire further, we shall find that distinct sympathetic actions are dependent on each of these peculiar sensibilities, all of which are roused into activity by this individual nerve. 1. Dashing cold water on the skin of the face or head, produces a deep inspiration. 2. The surface of the eye being irritated, causes convulsive action of the orbicularis oculi, and draws tears from the lachrymal gland. 3. Tickling the inside of the nose excites the whole apparatus of the respiratory muscles into violent action, to produce sneezing; and in this case we may observe that the actions of a numerous class of muscles are arranged so as to direct and concentrate the expired air to sweep off the offending cause from the nostril. 4. Sapid bodies being applied to the tongue, cause an increased flow of saliva to the mouth, and draw into action the organs of mastication.

Cases have occurred* which prove that, when the fifth pair is destroyed, the sensibilities being lost, the muscles cease to produce their sympathetic actions ; so that the eye

* See "Exposition of the Nervous System."

may be pressed with the finger without causing winking, and the inside of the nose may be tickled without sneezing. Yet it is observed that patients, in these circumstances, can shut the eyelids, or draw the other muscles of the face into action, by voluntary effort.

It will now, perhaps, be allowed that we have made a considerable step towards connecting anatomy with the "principle of sympathy." We may not, indeed, be able to explain why a certain kind of sensibility produces a certain sympathetic action, for that must depend upon the sensorium; but we have traced the individual nerve which conveys the *sensation* to the brain. Take sneezing, for example; we know that it is the fifth pair which receives the impression, and transmits it to the brain; and we further see that there follows the sensation, so produced, a peculiar excitement, drawing certain muscles into action. We observe that the muscles excited are of the class called respiratory; and if we find that, when certain nerves are entire, the series of actions above alluded to is produced,—but that, when they are cut across, no action takes place, however the nerve of sensation may be excited,—is it not fair to conclude that they are respiratory nerves? Thus it is by observing the manner in which certain nerves are brought into combination in each sympathetic action, that we may discover the motor nerves to which the impression was conveyed through the sensorium.

It may be seen that, belonging to respiration, there are certain sensations which excite peculiar sympathetic actions, for the protection of this important function. It has not been asserted that the organs of respiration are peculiar in this respect: indeed, several instances have been stated, which show that the "principle of sympathy" extends over the whole body; and the only *peculiarity* of the respiratory apparatus is, that there exists a class of nerves destined for its functions, distinct from those which supply other parts.

If we attend particularly to the sensibilities which influence the act of respiration, we find that nature has guarded this function with a sensibility which is at all times awake. Under ordinary circumstances, it controls the complicated actions of breathing, without the cognizance of the will; and it becomes the object of our perception only when danger to the function is impending. No sooner, however, is this the case, than the sensation we call *anxiety* is excited; by which the respiratory muscles are sympathetically roused to their utmost exertion. We have frequent opportunities of observing the heaving of the chest and shoulders, the gasping and contractions of the throat, the spasmodic twitchings of the face, the energy that pervades the whole frame, in those who suffer from obstruction in the windpipe, or die from hæmorrhage. The sensation seems to have its origin in the heart and lungs: being conveyed to the sensorium, it produces those phenomena through the medium of the respiratory nerves.

Through this sensibility, the heart and lungs are so combined, that, in all the variations to which they are liable, they are still found to correspond; the accelerated action of the one being directly followed by the excitement of the other. If there be an increased activity in the heart, it is necessary, for the circulation of the blood, that the apparatus moving the lungs should be accelerated in a corresponding degree: and, as the lungs themselves are passive, the influence must be sought for in those muscles which move the chest, the throat, and the nostrils.

In various powerful affections of the mind, there is a sensation referred to the breast, the existence of which is manifested by external signs. Writers on Ethics have classed *emotions* separately from the intellectual states of the mind, on account of the peculiar "vividness of feeling" which belongs to the former.* It would appear that simple consciousness is too dull a medium for these affections of the mind to be impressed through it alone; and there is added an excitement of the frame, which gives intensity to the feeling. There is a certain turbulence produced in the action of the heart and the dilatation of the chest, and in the contractions of the throat and face, whenever the mind is intensely affected with emotion: thus, for example, in terror, the heart beats with force against the ribs; "there is a spasm on his breast; he cannot breathe freely; the chest is elevated; the muscles of the neck and shoulders are in action; his breathing is short and rapid; there is a gasping and convulsive motion of his lips, a tremor on his hollow cheek, a gulping and catching of his throat." The explanation of these phenomena seems to be this:—The nerves of respiration have been influenced by the condition of the mind to produce these actions; this disturbed state of the heart and respiratory organs is quickly referred to the mind, through that sensibility which we formerly said watches over them: and thus a sensation is produced, very nearly resembling that which accompanies threatened suffocation.†

This view of the matter illustrates how corporeal sensations

* Brown's "Lectures on the Philosophy of the Human Mind," vol. iii. p 29.

† For further illustrations of this subject, the reader is referred to the second edition of Mr. Bell's Essays on the Anatomy of Expression.

are reflected to the mind, to render us more vividly alive to its emotions; and it also shows that the mind may rouse certain parts of the body into activity, independently of a preceding sensation. In these instances, sensation is not the cause, but the consequence, of the particular actions already produced in the body.

According to Dr. Alison, in adopting Mr. Bell's views,

"We should be obliged to suppose that there are bound up, in what is commonly called the olfactory nerve, one nerve destined for the perception of nauseous smells, and connected with the nerves of the abdominal muscles and diaphragm,"—(and the stomach, &c. if he supposes the act of vomiting;)—"one for the smells that cause faintness, connected with the nerves of the heart; and one for savoury smells, connected with those of the salivary glands. And in the fifth pair of nerves we must suppose that there are bound up, one nerve for the perception of cold applied to the face, and connected with the phrenic and intercostal nerves, by which the act of inspiration is performed, and also with the cutaneous nerves of the whole surface, by which a general constriction of the capillaries is produced; one for the perception of irritations of the nostrils, connected with the phrenic and lower intercostals and lumbar nerves, by which the act of sneezing is performed; and one for the perception of pain in the eyeball, cheeks, or teeth, possessing little or no connexion with the respiratory nerves; and so in other instances."

If, indeed, we expected to see all obscure phenomena explained,—the effects, for example, of odours giving pleasure or pain, or exciting disgust and nausea: if all those singular nervous affections, which are variously exhibited in different individuals, had been attempted to be accounted for, by this new system of the nerves, we should have thought of Mr. Bell's observations as Dr. Alison does. But we have remarked that he most resolutely avoids entering on subjects of this kind, which promise no successful results. He has limited us to the observation of certain facts: he has taught which nerves convey sensation from the part to the sensorium: he has shown by what nerves the impression proceeds from the sensorium to the muscles, combining them into simultaneous operation. But he has not attempted to explain anatomically what connexions are formed betwixt the roots of the sensitive nerves and the muscular nerves, in what is called the sensorium. We have heard Mr. Bell propose it as a question in his Lectures, how far the motions of parts, excited by titillation or irritation, depend upon the connexions of the nerves in the body, and how far on the connexions of the nerves in the brain. For example, when a creature continues to breathe without its head, or when an acephalous fœtus breathes without cerebrum or cerebellum, is there not a connexion between the heart and lungs and the muscles of respiration, without the intervention of the brain? He illustrated this question by mentioning, on the other hand, the muscles of the eye acting under the irritation of the eye, as a proof that the brain forms a part of this circle of relations; but the excitement of the heart and the intestines, contracting upon being distended, as other instances of the connexion being formed without the intervention of the brain. Dr. Alison is not aware of the care with which Mr. Bell has impressed in his Lectures, that there is a whole system of nerves, of which the powers are not yet ascertained,—those called the *sympathetic,*—which may, at some future time, be made to resolve these difficulties.

If we have succeeded in proving that the "principle of sympathy," as it is presented to us by Dr. Alison, is not inconsistent with Mr. Bell's views, but is confirmed by them, we may expect, as a natural consequence, that Dr. Alison's objections to the classification of the nerves are nugatory.

The principal cause of his objecting to the system of the respiratory nerves, is founded on his not having separated in his mind the nerves of sensation from the nerves which command muscular action. Thus, because tickling the nostril excites the action of the muscles connected with respiration, he wonders why the branch of the fifth pair, within the nostril, is not included among the respiratory nerves. (P. 200.) We have already proved that nerves of sensation produce various sympathetic actions, by their communication with the sensorium. The nerves of respiration are explained to be those which are distributed on a peculiar class of muscles, to control the actions connected with breathing. The nerve of the nose possesses an influence over the respiratory actions only in an indirect and incomprehensible manner, by its connexion with the sensorium. If we desire to know which nerves produce the phenomena, we must look to the muscles which are called into action: these indicate which nerves were excited, because we know the nerves that go to supply these muscles. We should never think of looking among the nerves of *sensation,* to discover those destined to effect any *motion.* Here, then, appears to be the error of Dr. Alison: he has looked for respiratory nerves among nerves of sensation, when he might have known that the nerves of sensation form a distinct class in Mr. Bell's system; and he might have found that the fifth pair is included in that class. The peculiarity of the respiratory nerves consists in their being motor nerves, which supply the muscles employed in the actions of breathing, bestowing upon these muscles those peculiarities which distinguish them from all others, and combining them to act with one simultaneous effort.

It is necessary for us to remember that the respiratory nerves, in their course, are connected with nerves belonging to the other classes; so that the parts to which they go may appear to possess, through them, more endowments than one. If we find an organ, which is supplied by respiratory nerves, possessed with a peculiar sensibility, as the epiglottis, we are to ascribe the sensibility to the sensitive part of the spinal nerve, with which the respiratory nerve is bound up in one sheath; but the power of drawing the small muscles around the epiglottis into action, belongs to the respiratory nerve.

Let us take an example to prove that anatomy points out to us a difference between the nerves of muscular power. For this purpose, let us select the fifth pair and portio dura of the seventh pair; both of which are nerves bestowing muscular motion. The fifth pair is a compound nerve, resembling the spinal nerves, arising by two roots: the nerves proceeding from one root bestow sensibility to the head, while the other bestows power to the muscles of the jaw. The anatomical character of the fifth pair is, that it is distributed to almost every part of the head. It can even be traced most distinctly sending its branches to the muscles of the face. We have said that the fifth pair is a muscular nerve; yet these branches, which it sends into the muscles of the face, are not the motor nerves of the face: they possess no direct power of exciting these muscles to contraction. It would appear from this alone, that these muscles are of a class which require their functions to be bestowed by a nerve different from a common one. This leads us to observe what are the distinctions between the muscles of the jaws and those of the face, to ascertain whether their functions be different. The muscles of the face are subject to changes depending on the quiet or excited condition of the apparatus of respiration. Involuntary actions in the muscles of the eyelids, of the nostrils, and cheeks, are the consequences of the respiratory organs being violently roused. These muscles are much affected in certain passions of the mind, when we observe the whole respiratory organ moved. There is another peculiarity regarding these muscles: in certain conditions they are placed beyond the influence of the will, as in continued breathing, yet they are also subject to the will, as we observe in speech; and we must not omit to notice that the involuntary actions necessary for breathing, proceed at the same time that these muscles are employed, under the direction of the will, for other purposes. All these circumstances prove that the muscles of the face are endowed with properties which require a nerve different from a common nerve. Accordingly we find that the portio dura, the respiratory nerve of the face, comes from a source in the brain different from that of the fifth pair; that, at its origin, it is close to other nerves, which may be proved to possess similar endowments with it; and that it takes a course different from the fifth pair, for the apparent purpose of combining its branches with the respiratory nerves. Are these not fair grounds for arranging this nerve (the portio dura) among a distinct class from the symmetrical nerves, and giving it a name corresponding to its character?

We shall here notice briefly another of the objections stated by Dr. Alison. He suggests (p. 198,) that the ninth pair of nerves ought to be included among the respiratory nerves, because the tongue is combined, during speech, with the apparatus of breathing: he thinks also that the branches of the fifth pair, which control the motions of the lower jaw, ought to be joined to this class. We conceive that speech may be proved to be a compound operation,—a superadded function, connected with the organ of breathing. If we endeavour to analyse articulate language, we may observe that one part consists of simple inspiration and expiration of the air: the motions of the tongue and of the jaws, and of other parts, may be separately considered as functions superadded to breathing: it is through these motions that the air is split and divided, so as to constitute articulate language. Those actions which produce breathing, whether forcibly expelling the air or gently expiring it, or drawing in the breath softly or with emphasis, form one distinct organ. Those actions which serve to modulate and divide the current of air, may be considered distinct and by themselves. This view of the subject may perhaps afford an explanation why nerves of different classes are seen weaving their branches in the same part.*

It is apparent that the functions of every organ require very accurate investigation, before we can judge of the effects of the nerves. It is easy to frame objections—for the term ***respiratory*** is not sufficiently definite to those who have not studied the functions of the respiratory organs; and the word only serves to express the necessary relation which nerves so called have with the actions of respiration.

We, who are pleased with the simplicity and the interest which these views give to the nervous system, may be allowed to express a regret that an ingenious physiologist, conversant with many of those authors who have written on the *theories*

* See Harris's Hermes, p. 319.

of the nerves, should present us their *anatomy* in the same intricacy with which it has been hitherto enveloped.

Let us mark the advantages of physiology grounded on the close observation of Anatomy. First, to have shown the composition of the spinal nerves; that the whole range arising from the spinal cord, having ganglions upon their roots, are sensible nerves; and that the anterior roots are muscular nerves; Secondly, that the fifth pair in the head is of the same composition with the nerves of the spine; Thirdly, that, as this nerve corresponds in structure with the spinal nerves, so it corresponds in function; Fourthly, to have distinguished the nerves of the face into two classes, and shown that they possess distinct endowments; Fifthly, to have traced a set of nerves exclusively distributed upon the muscles of respiration; Sixthly, to have shown, by comparative anatomy, that these nerves do not exist, except when the act of respiration is to be performed:—These form altogether a most brilliant proof of the importance of anatomical investigation.

We have heard some gentlemen say that these new views are very ingenious and very curious; but they have asked, of what use are they in the practice of medicine and surgery? We may reply, that it is impossible, in the early stage of any discovery, to foresee how far its influence may extend. But is it nothing to have proved that palsies, which were supposed to depend on the condition of the brain, may arise from local causes quite independent of that organ, and comparatively of little danger?—To have pointed out to the surgeon the difference between a motor nerve and one of sensation,—and the consequent impropriety of cutting them indiscriminately in cases of tic douloureux, as formerly? No surgeon, acquainted with these new views, in operating on the face, would divide the portio dura, in the expectation that its functions would be performed by branches of the fifth pair. Neither would he hazard the destruction of the eye, when operating on tumors in the vicinity of those branches of the portio dura which pass to the eyelids, by their division.

In conclusion, we would say that it is to be regretted, with regard to papers such as we have been endeavouring to criticise, that the general reader, who may not perhaps be very intimately acquainted with the subject, is more apt to rely on the character of the writer, than to weigh critically the value of his arguments.*

* We are indebted for this paper to "a Windmill-street Pupil:" from the manner in which it is executed, we hope he will be induced to continue his contributions.

*A Treatise on Diet: with a View to establish, on practical grounds, a System of Rules, for the Prevention and Cure of the Diseases incident to a Disordered State of the Digestive Functions.* By J. A. Paris, M.D. F.R.S. Fellow of the Royal College of Physicians, &c. &c.—8vo. pp. 307. London: T. and G. Underwood. 1826.

(Concluded from page 166.)

*Of Drinks.*—Whatever may be the contrariety of opinion entertained by different physicians upon the subject of the solid articles of our diet, we shall find it equalled, or perhaps exceeded, by the discrepant views that are maintained relative to the quantity and quality of the liquids in which we ought to indulge, either for the purpose of preserving health, or of lessening the severity of disease. In order that the practitioner may prescribe with advantage the quantity of fluid that should be taken, he must first be acquainted with the aliment upon which the patient lives; for different aliments require different quantities of liquid to assist their chymification. That he may recommend the fluid most suitable in quality, he must deliberately consider not only the bodily but mental condition of the subject. Notwithstanding, however, the necessity of frequently abandoning abstract rules to meet individual peculiarities, such general directions may be given as will be of considerable service in practice.

"By drinking *before* a meal, we place the stomach in a very unfit condition for the duties it has to perform. By drinking *during* a meal, we shall assist digestion, if the solid matter be of a nature to require it; and impede it, if the quantity taken renders the mass too liquid. Those physicians, therefore, who have insisted upon the necessity of a total abstinence of liquid during a meal, appear to have forgotten that every general rule must be regulated by circumstances. The best test of its necessity is afforded by the sensations of the individual, which ought not to be disregarded merely because they appear in opposition to some preconceived theory. The valetudinarian who, without the feeling of thirst, drinks during a meal, because he has heard that it assists digestion,—and he who abstains from liquid in opposition to this feeling, in consequence of the clamour which the partisans of a popular lecturer have raised against the custom,—will equally err, and contribute to the increase of the evil they so anxiously seek to obviate. Dr. W. Philip has stated a fact, the truth of which my own experience justifies, that 'eating too fast causes thirst; for, the food being swallowed without a due admixture of saliva, the mass formed in the stomach is too dry.'" (P. 118.)

In more than one instance we have known much temporary inconvenience, if not permanent mischief, caused by a rigid adherence for a considerable length of time to the arbitrary and peremptory commands which have been given, not to permit any fluid to be taken during the meal. Many persons, it is true, can dine without drinking, and suffer no distress from the privation; but in a majority of cases, in which this plan is attempted, we shall find that the patient takes his food without his accustomed relish, and that the process of digestion is more painfully performed than if a *moderate* quantity of liquor were allowed. The observations, then, of Dr. Paris are rational and correct. He lays down no dogmatical rule, by which every patient is to be guided, and by which every disease is to be relieved.

*Water* is considered to be unquestionably the natural beverage of man; " but (says Dr. P.) any objection against the use of other beverages, founded on their artificial origin, I should at once repel by the same argument which has been adduced in defence of cookery. We are to consider man as he is, not as he might have been, had he never forsaken the rude path of nature. I am willing to confess, that, 'the more simply life is supported, and the less stimulus we use, the better; and that he is happy who considers water the best drink, and salt the best sauce:' but how rarely does a physician find a patient who has regulated his life by such a maxim! He is generally called upon to reform stomachs already vitiated by bad habits, and which cannot, without much discipline, be reconciled to simple and healthy aliment. Under such circumstances, nothing can be more injudicious than abruptly to withdraw the accustomed stimuli, unless it can be shown that they are absolutely injurious." (P. 119.)

We must refer to the work itself for some observations upon the comparative salubrity of different kinds of water.

*Tea.*—No subject has excited greater contrariety of opinion amongst dietetic writers, than this common beverage. Some decry it as a poison; others extol it as a valuable medicine. By some, all the beneficial effects of tea are placed to the account of the water thus introduced into the system; and its evil effects are attributed to the high temperature at which it is drank.

" In order to understand the value of the different arguments which have been adduced in support, or to the disparagement, of this beverage, it will be necessary to inquire into its composition. Two kinds of tea are imported into this country, distinguished by the epithets *black* and *green*. Both contain astringent and narcotic principles, but in very different proportions; the latter producing by far the most powerful influence upon the nervous system. As the primary operation of every narcotic is stimulant, tea is found to exhilarate and refresh us, although there exist individuals who are so morbidly sensible to the action of certain bodies of this class, that feelings of depression, accompanied with various nervous sensations and an unnatural vigilance, follow the potation of a single cup of strong tea; while others experience, from the same cause, symptoms indicative of derangement of the digestive organs: but these are exceptions, from which no general rule ought to be deduced. The salubrity of the infusion to the general mass of the community, is established by sufficient testimony to outweigh any argument founded on individual cases. It must, however, be admitted that, if this beverage be taken too soon after dinner, the digestion of the meal may be disturbed by the distention it will occasion, as well as by its influence as a diluent; the narcotic and astringent principles may also operate in arresting chymification: but, when a physician gives it his sanction, it is with the understanding that it shall be taken in moderate quantities, and at appointed seasons. When drunk four hours after the principal meal, it will assist the ulterior stages of digestion, as already explained, and promote the insensible perspiration; while it will afford to the stomach a grateful stimulus after its labours. With regard to the objection urged against its use on the ground of temperature, it will be only necessary to refer to the observations which have been already offered upon this subject. In enumerating, however, the advantages of tea, it must not be forgotten that it has introduced and cherished a spirit of sobriety; and it must have been remarked by every physician of general practice, that those persons who dislike tea frequently supply its place by spirit and water. The addition of milk certainly diminishes the astringency of tea; that of sugar may please the palate, but cannot modify the virtues of the infusion." (P. 129.)

*Coffee* exerts a different species of action upon the nervous system to that produced by tea, although it is difficult to define the nature of this difference.

" If taken immediately after a meal, it is not found to create that disturbance in its digestion which has been noticed as the occasional consequence of tea: on the contrary, it accelerates the operations of the stomach, and will frequently enable the dyspeptic to digest substances, such as fat and oily aliment, which would otherwise occasion much disturbance. The custom of taking coffee immediately after dinner, as so universally practised by the French, no doubt must counteract the evil effects which the peculiar form of their diet is calculated to produce. Coffee, like tea, has certainly an antisoporific effect on many individuals; it imparts an activity to the mind which is incompatible with sleep: but this will rarely occur if the beverage be taken several hours before our accustomed period of repose. It seems to be generally admitted, that it possesses the power of counteracting the effects of narcotics; and hence it is used by the Turks with much advantage, in abating the influence of the inordinate quantities of opium they are accustomed to swallow. Where our object is to administer it as a promoter of digestion, it should be carefully made by infusion; decoction dissipates its aroma. The addition of milk is one of questionable propriety; that of sugar, or rather sugar-candy, may be allowed.* I have known some persons who have never taken this beverage without suffering from acidity in the stomach: where this happens, the practice must be abandoned." (P. 130.)

*Chocolate* and *cocoa* are not be considered as desirable articles of diet, in most instances.

*Soda water.*—The remarks upon this fashionable beverage, to which the caprice of the day attributes many virtues it certainly does not possess, whilst its frequent ill effects are almost entirely overlooked, are brief, but pertinent.

"The modern custom of drinking this inviting beverage during or immediately after dinner, has been a pregnant source of dyspepsia. By inflating the stomach at such a period, we inevitably counteract those muscular contractions of its coats which are essential to chymification. The quantity of soda thus introduced scarcely deserves notice: with the exception of the carbonic acid gas, it may be regarded as water; more mischievous only, in consequence of the exhilarating quality inducing us to take it at a period at which we should not require the more simple fluid." (P. 132.)

*Fermented liquors.*—Dr. Paris ridicules the abuse which has been so indiscriminately indulged in by many authors, against even the moderate use of this class of fluids. He believes, and we think correctly, that there exists no evidence to prove that a *temperate* use of good wine, when taken at seasonable hours, has ever proved injurious to healthy adults. Upon the subject of *beer* he observes, that he believes the fraudulent adulteration of porter to be much exaggerated; and that, at any rate, such adulterations are not carried on in the cauldrons of the brewer, but in the barrels of the publican. The origin of the beer called *entire* is thus explained:

"Before the year 1730, the malt liquors in general use in London were ale, beer, and twopenny; and it was customary to call for a pint, or tankard, of half-and-half,—i. e. half of ale and half of beer, half of ale and half of twopenny. In course of time

* "Coffee has been often imitated by the torrefaction of various grains. In the 'Fourth Century of Observations,' in the 'Miscellanea Curiosa,' we find a critical dissertation on the coffee of the Arabians, and on European coffee, or such as may be prepared from grain or pulse. Dillenius gives an account of his own preparations made with peas, beans, and kidney-beans; but says that made of rye comes nearest to true coffee, and was with difficulty distinguished from it. This fact is curious, inasmuch as a spurious coffee has been lately vended, which is nothing more than roasted rye. The article is well known, under the name of 'Hunt's Economical Breakfast-powder.'

it also became the practice to call for a pint, or tankard, of *three-threads,*—meaning a third of ale, beer, and twopenny; and thus the publican had the trouble to go to three casks, and turn three cocks, for a pint of liquor. To avoid this inconvenience and waste, a brewer, of the name of Harwood, conceived the idea of making a liquor which should partake of the same united flavours of ale, beer, and twopenny. He did so, and succeeded, calling it *entire*, or *entire butt,* meaning that it was drawn entirely from one cask or butt; and, as it was a very hearty and nourishing liquor, and supposed to be very suitable for porters and other working people, it obtained the name of *Porter*."

We pass over the " Estimate of the Nutritive and Digestible Qualities of different Aliments," and the observations upon " the Periods best adapted for Meals, and on the Intervals which should elapse between each," as not containing any thing of particular interest.

Part III. " of Indigestion."—Contrary to the nomenclature adopted by Dr. PHILIP, our present author considers the terms *dyspepsia* and *indigestion* to be synonymous. Indigestion is defined by Dr. Paris " to be a primary disease, in which one or more of the several processes by which food is converted into blood are imperfectly or improperly performed, in consequence either of functional aberration or organic lesion." It is suspected that this definition may be thought to have too comprehensive a signification: but, says the author,

" I may observe, that, however extensive may be the series of symptoms which are thus included under one general head, they will afford, when viewed collectively, sufficient evidence of their relation with the digestive process; although, on a loose and hasty observation, they may not present any general principle of dependency and connexion: if they appear disunited, let the practitioner suspect that he has never viewed them with sufficient reference to that physiological harmony which subsists between the organs in which they arise. Acidity of stomach and urinary depositions are equally indicative of deranged digestion; but the mind that is not acquainted with the relations of the stomach and kidneys, or with the connexion which subsists between the formation of perfect chyle and the discharge of natural urine, will not be disposed to arrange symptoms, so apparently remote in their alliance, under one common head. There are many sympathies subsisting between different functions, which are not perceptible as long as the general balance of health is preserved: this is remarkably the case with the skin and stomach; but, the moment this healthy equilibrium is destroyed, the sympathies become apparent. The physiologist, therefore, without an acquaintance with the body in its morbid states, must remain ignorant of some of the more important circumstances of the animal economy. The same reasoning applies to the study of natural philosophy: the discovery of the existence of an electric fluid could never have been made, had the natural conditions of matter, with regard to this agent, remained unchanged. The basis of all chemical research is founded upon the same principle: decomposition, and the development of the elements of bodies, are effected by overturning the affinities by which they are naturally combined. These observations are introduced in order to warn the practitioner not to deduce any conclusion against the existence of certain sympathies, on the ground of their not being apparent in a state of health. In a practical point of view, I consider the classifications of the nosologist as of very little utility: they have no solid foundation in nature, but are entirely the work of human reason,—artificial contrivances, for the purpose of assisting us in the acquirement and retention of knowledge. Such an avowal will sufficiently explain the motive which has induced me to throw off the trammels to which I might have been expected to conform." (P. 214.)

Physicians of the present day are justly believed to accuse the alimentary functions of offences which should be charged on other organs. Dr. Paris is sceptical as to the existence of any malady which is entitled to the specific appellation of "dyspeptic phthisis;" a term particularly employed by Dr. W. Philip.

"A person having tubercles in the lungs may have his life protracted for many years, by judicious management, and by avoiding every exciting cause which might kindle the spark into flame, by keeping the circulation in check, and promoting the healthy action of the secretions. On the contrary, the fatal termination may be equally accelerated by want of care; and, above all, by creating a permanent disturbance in the digestive functions. If Dr. Philip designates a latent disease, thus kindled into activity, '*dyspeptic phthisis,*' I have no objection to the term: we are no longer at issue, our difference of opinion is not essential; it is an affair of words, and of words only." (P. 235.)

The *symptomatology* of indigestion is sketched with brevity, but with accuracy. No dyspeptic can peruse it without recognising the fidelity of the description. Our author, perhaps, in common with most writers who treat upon this distressing affection, lays too little stress upon the pain that is frequently felt by dyspeptic patients down the arms, particularly near the insertion of the deltoid muscle, which is generally also accompanied by a numbness of the fingers and weakness of the whole arm, especially on the left side. These symptoms are very common in patients who have for some time laboured under indigestion, and require especial mention, as by many authorities they are looked upon as pathognomonic signs of some organic disease of the heart. In order that the practitioner may conscientiously perform his duty to the dyspeptic patient, the frequent examination of the fæces is insisted upon; and, as it may occasionally happen that the preservation of them is attended with inconvenience, "it is worthy of notice, that a table-spoonful of sweet oil poured over them, by investing the surface with a film, effectually prevents evaporation, and the consequent stench they might otherwise occasion."

The *indications of cure* in indigestion must be derived from the previous habits of the patient, and the origin and seat of the disorder. That practitioners frequently err by requiring from dyspeptic patients a strict adherence to particular modes of living and management, without reference to former habits, is very certain. Hence the propriety of the observations made by Dr. Paris, in discussing the general plan to be adopted by the practitioner in the treatment of this ever-varying and perplexing malady.

"He must lay down a system of rules for his patient, by which the remote causes of his complaints may be removed; but, in his attempts to reform bad habits, he must be careful to avoid all abrupt transitions, except in those circumstances which have no direct influence upon the vital powers of the body. I should, for instance, be very cautious how I withdrew spirituous stimulants, although I might be well satisfied that the indulgence of such potations had given origin to the disease; but I should not feel any hesitation in at once withholding every species of pastry, or other indigestible matter, without reserve. Upon the same principle, we should gradually diminish the number of meals, where they have exceeded the proper limit, adopting them with skill and caution to the fluctuating circumstances of the patient. The same observation applies to exercise. Nothing would be more injudicious than to expose the invalid, debilitated by sedentary habits, to the effects of sudden and protracted exercise; nor should the person, whose habits have been laboriously active, be abruptly restricted to an irksome state of indolence. The discipline, in such cases, must be graduated according to the previous habits of the patient; to his age, strength, and the nature of his disease. Exercise can never prove salubrious, if it be followed by fatigue. Mr. Abernethy has prescribed to his patients a set of rules, which I shall take the liberty of quoting in this place, in order that I may offer such observations upon their value as my own experience has suggested. '*They should rise early when their powers have been refreshed by sleep, and actively exercise themselves in the open air till they feel a slight degree of fatigue.*' Upon this first rule, I am disposed to make the following comment:—Although we must all agree in the advantages of early rising, yet, in dyspeptic cases, I have frequently known the disease greatly aggravated by the patient suddenly changing his habit, with regard to the hour of rising; and that, if he becomes the least fatigued before his morning repast, he remains languid and uncomfortable during the rest of the day. A long walk before breakfast, unless the person has been accustomed to the practice, will frequently produce a fit of indigestion. I have already observed, that it is advisable to allow an interval to pass before we commence the meal of breakfast; and, where the weather and circumstances will permit it, this interval should be passed in the open air, but the body should not suffer the least fatigue." (P. 260.)

Dr. Paris observes, that it has frequently been considered doubtful whether the acidity which occurs so frequently in the stomachs of dyspeptic patients, arises from the fermentation of the food, or from a vitiated state of the gastric secretion. He is of opinion that it may arise from either cause, and makes the following observation, which, if correct, is of much practical importance.

"In cases of imperfect chymification, it would appear more generally to depend upon the matter generated by the food; for it is instantly relieved by a dose of carbonate of soda: but where it is symptomatic of some disease in a distant organ, as in that of the uterus, it would seem to be connected with an acid state of the gastric juice, and is not relieved by the administration of alkalies. I am not aware that this distinction has ever been established, but I am so well satisfied of its truth, that I have in several cases been led, from this circumstance alone, to distinguish between primary and symptomatic affections of the stomach. Unrelenting and continued cardialgia ought to lead us to suspect disease in some other organ; although I am not prepared to state that it is never the effect of a primary affection of the stomach." (P. 264.)

The efficacy of alkaline remedies, and particularly the carbonate of ammonia, in removing acidity of the stomach, cannot be doubted; but more is frequently expected from antacid remedies than they are capable of performing. Permanent relief can only be obtained by a change of food; by abstinence from vegetables, if the patient can submit to it without disgust. To relieve the flatulence with which dyspeptics are occasionally so much tormented, Dr. Paris recommends us rather to calm the irritability of the bowels, than to have recourse to the use of carminatives for the purpose of expelling the flatus. He conceives that "it is not the presence of gas in the intestinal canal, but the irritability of the intestines, which renders them impatient of the slightest stimulus of distention, that occasions the distress so common to dyspeptic invalids." Small doses of extract of hyoscyamus, combined with two grains of ipecacuan, are recommended as likely to relieve attacks of flatulence, which have resisted the ordinary modes of treatment. If the dyspeptic disease is connected with irritation of the duodenum, the vinum colchici is considered highly useful, taking care to accompany its exhibition with occasional laxatives. In every stage of dyspepsia, active purgation is said to be extremely mischievous. We grant the truth of the doctrine in most cases; but we should be inclined to state it with some qualification. We are sometimes called upon, at the commencement of dyspeptic attacks, to clear the bowels by active purgatives, however improper the repetition may be in the latter stages of the complaint. The fashionable remedy of the day, in all classes of society, and in all classes of disease, the white mustard seed, is considered to be a useful medicine in several morbid states of the intestinal canal; but, according to the experience of the author, it is serviceable only in such cases as are marked by alimentary torpor, as may be gathered from the extract given in our last Number.

The medical student will consult this volume of Dr. Paris with much advantage, although, perhaps, the well-informed practitioner may consider it to fall very short of a full and elaborate treatise upon the various subjects which are considered. That it contains but little novelty, cannot in justice be urged as a complaint against the author, as it appears to have been his intention rather to discuss what has been said by others, than to hazard any original speculations, or to make known any new facts derived from his own labours.

---

*A Case of Melanosis, with general Observations on the Pathology of this interesting Disease.* By Thomas Fawdington, Member of the Royal College of Surgeons, London; and one of the Surgeons to the Manchester Lying-in Hospital. With Plates. —8vo. pp. 49. London: Longman and Co. 1826.

To swell a single case into a volume, must be allowed to require considerable ingenuity: it appears, indeed, that the present paper was originally destined for insertion in some of the periodical Journals, "but, (says the author,) owing to the suggestions of some of my professional friends, in whose judgment I am inclined to place great reliance, it has assumed the present form." We can only say that, in our humble opinion, his friends gave him very bad advice, as the circulation which the expedient he abandoned would have given to his pamphlet, would have been incomparably greater than he can now hope for. The case is a very well marked one of Melanosis, and the plates in illustration are executed in a very creditable manner; indeed we may be allowed to say, without offence to the author, that they constitute by far the most important part of the volume. These, unfortunately, we cannot communicate to our readers; but we shall lay before them some details, of sufficient interest to recompense the trouble of perusing them: they relate to the dissection of the eye after its extirpation,—to the minute structure of melanosis,—and to its chemical composition.

" *Melanosis of the eye.*—" A section of the eyeball discovered a black pultaceous tumor, occupying more than one half the interior of the globe, in the situation of the vitreous humour; of which last-named part no trace could be discovered. There were two cavities or cells, filled with a brownish red fluid; one situated at the side of the tumor, the other anterior to it and behind the lens. No trace of the vitreous cells could be discovered. The tunica choroides was entire, and could easily be drawn up from the sclerotica, except at one point towards its superior and internal part, where it ceased to be distinguishable from the general mass of the tumor. The sclerotica was here reduced to an extreme degree of tenuity, and had a split appearance. The retina was quite detached from the choroid by the interposition of the disease, and laid folded across the globe, forming a kind of septum between the black mass and the larger of the two cavities containing the brownish red fluid. The lens was opake, and of a yellow hue; the capsule thickened, but partially transparent: a fold of retina covered the posterior capsule, or that fold of the hyaloid which lines the vitreous fossula for the lodgment of the lens. The ligamentum ciliare was distinct, and some ragged portions of membrane at the margin of the lens and posterior to the iris, which was perfect, showed a remnant of the ciliary processes. The optic nerve, where it had been divided at the time of the operation, appeared to be sound." (P. 7.)

*Intimate structure of Melanosis.*—" On inspecting more closely the product of melanosis, it was found to possess an appearance very similar to that which the contents of a decaying lycoperdon, or common puff-ball, would present, if rendered cohesive by the addition of a small proportion of liquid. It had a deep red-brown or chocolate colour; was slightly fibrous in texture; and, when agitated in water or spirit, and suffered afterwards to rest, a part fell down, as a pulverulent sediment, having the colour and opacity described. The water received a deep tinge from it; the spirit was hardly coloured. This substance, in its most compact form, showed a tenacity equal to that of the brain; and yielded, by expression, a small portion of a reddish fluid, intermixed with fragments of the texture alluded to; but it had become reduced to a thin blackish or red-brown paste, where the softening was completed. The softening was most decided in the centre of the largest tubera, and in these the inner circumference of the cysts was fringed with flocculi of the melanose material, which were connected with it by means of a very fine cellular tissue; and this latter formed the bond of union between the cyst and its contents, under all circumstances. By the same medium the external surface of the cysts was united to the parts in which they were situated; and these, in a general way, admitted of being easily detached. The cysts did not present the slightest trace of vascularity, nor was there any visible turgescence in the vessels of the enclosing textures. M. Breschet has been at some pains to ascertain whether the melanose tubercle was truly organised; and with this view he threw into the veins and arteries of the contiguous parts some of the finest and most diffusible injections, without discovering any continuity of vessel between the cyst and the substance it contained, or any organisation in the latter. He states the results of these experiments in these words:—'Mon injection n'a fait que dénoncer des vaisseaux sur la membrane d'enveloppe et quelquefois il s'en est épanché dans la cavité qui s'est mêlée à la sub.tance morbide.'" (P. 25.)

*Chemical analysis of Melanosis.*—" From an analysis which he made of it, M. Barruel, a French chemist, thinks that the substance of the tumors in melanosis is a deposition of the colouring matter of the blood, and of fibrine, each under a particular modification, and forming three distinct fatty substances. The first soluble in alcohol at a moderate temperature, and disposed to crystallise in brilliant scales; the second is soluble in alcohol at a boiling heat only; the third is a fatty substance, in a fluid state at the ordinary temperature of the air, of a reddish colour, containing a large portion of the phosphates of lime and iron.

" From a portion of the softened matter, after it had been kept some time in spirit, Dr. Henry obtained the following results, which, through his politeness, I am enabled to give in his own words:

" 1st. By filtering through paper, much of the colouring matter remained on the paper, and the colour of what passed through was much less intense.

" 2d. Boiling does not destroy the colour, nor even when a little caustic potash has been added.

" 3d. It is not changed by acids, even when heated, except by nitric acid, which deprives it of its black colour, and turns it yellow.

" 4th. A stream of chlorine passed through the liquid destroys the black colour, and throws down light fawn-coloured flocculi.

" 5th. A few grains of corrosive sublimate stirred up with the fluid, precipitates the whole of the colouring matter, and leaves the supernatent liquid quite clear.

" 6th and 7th. Nitrate of mercury, and muriate of tin, produce the same effect, but more slowly.

" From these experiments it appears that the black matter is a peculiar secretion, analogous in some properties, especially in the 5th, 6th, and 7th, to the colouring matter of the blood. It would be necessary, however, to repeat and extend the experiments on a larger quantity of the fluid, and in a more recent state, before any just conclusion can be deduced respecting its nature." (P. 27.)

*Experimental Researches on the Influence exercised by Atmospheric Pressure upon the Progression of the Blood in the Veins, upon that Function called Absorption, and upon the Prevention and Cure of the Symptoms caused by the Bites of Rabid or Venomous Animals.* (*Dedicated, by permission, to his Majesty.*) *With an Appendix, containing the original Reports of* Baron Cuvier *and of* Professors Dumeril *and* Laennec, *to the Royal Institute of France, and to the Royal Academy of Medicine of Paris, &c. &c.* By David Barry, m.d. Knight of the Order of the Tower and Sword; Member of the Royal College of Physicians in London; first Surgeon to the Portuguese Army, Surgeon to the Forces, &c. &c.—8vo. pp. 175. London: Thos. and Geo. Underwood. 1826.

Of this work it is not our intention to do more than give a short notice. To enter into the discussion would require more time, and demand a greater space, than we can afford upon the present occasion. But we consider the subject so important in a physiological point of view, that we should deserve censure if we did not recommend its consideration to our professional brethren, and request them, in perusing it, to throw aside all preconceived notions and prejudices.

Dr. Barry introduces his work with a short Preface, in which he discusses, with much earnestness and considerable force, the necessity of making experiments upon living animals. From this Preface we extract the following passage:

"The examination of a quiescent machine can only *suggest* the use of its parts when they were all in movement. Well-directed experiment upon these same parts, actively employed in fulfilling their various functions, either confirms the suggestion, giving it the validity of a law, or at once destroys the whole fabric of a baseless theory. 'Unicum sæpe experimentum, integrorum annorum laboriosa figmenta refutavit.' (Haller, tom. i. Præf.)

"The wisest and the most virtuous men of the ages they lived in spent a large portion of their time in making experiments upon living animals. Those of Harvey were honoured by the presence of his sovereign, who, by that act alone, would have been entitled to a share of the immortality gained by the illustrious discoverer of the circulation.

"Those who have stated that Harvey made but few experiments, and that to these few we owe but little, should have read his works. In these they would have learned that an unlimited supply of animals was placed at his disposal, by the enlightened prince to whom he was physician. His own words are singularly applicable to these candidates for unscientific popularity—'Qui nihil nisi homines secant." (P. xi.)

Again, in the next page, he observes—

"They who inveigh most loudly against experiments upon living animals, and who affect an excess of sensibility, have never made any experiments themselves. They are contented with the exposition of what they, in their wisdom, suppose nature *ought* to do, instead of investigating what she actually does.

"Others talk of needless cruelty. If any useful knowledge is to be obtained by an experiment, none of the means necessary to arrive at this knowledge can be needless, and none else can be adopted without defeating the purpose aimed at: therefore, in useful experiments, there never is needless cruelty, or, in other words, unnecessary pain, inflicted." (P. xiii.)

We fully agree with the general tenor of our author's sentiments upon this subject; but we do conceive that experiments of the most unnecessary and revolting kind have been, and are, too frequently put in practice by pretenders to physiological fame; that they have been multiplied still more wantonly, and the most obvious and established truths doubted merely to give the sceptic an opportunity of proving their truth by a useless display of practical anatomy upon some miserable animal. Against these practices we do most earnestly protest; but we are not so squeamish as to turn aside from experiments such as those which Dr. Barry has instituted, and are well disposed to give him all due credit for their originality, as well as for their ingenuity.

Our readers will observe that Dr. Barry's work consists of two Memoirs, and an Appendix containing the original documents presented by the different medical committees, to whom these Memoirs were entrusted for examination in France. The object of the first Memoir is to prove, from the anatomical structure of animals, as well as from direct experiment, first, the powers by which the blood is propelled through the veins to the heart; secondly, the comparative velocity in moving through the veins and arteries; and, thirdly, that the constant supply of blood to the heart cannot depend solely upon the causes to which it has hitherto been ascribed.

From the reasonings and experiments adduced in this first Memoir may be deduced the origin of the second Essay, on Absorption, which is in fact a corollary from the seventh proposition of the first Essay, which runs thus:

"That the lymph and chyle must be sucked up towards the chest, through the direct communications which the vessels peculiar to these fluids have with the subclavian and other veins. The question of absorption, therefore, which has hitherto puzzled physiologists so much, may now be considered as decided; for it is clear that the open mouth of a vein, or of any other vessel, having the same kind of communication with the thoracic pumps, must absorb in direct proportion to the sucking power applied to it, and to the pressure exercised upon the matter to be absorbed." (P. 37.).

If, says Dr. Barry, this be well founded, so ought the following corollary—" That the application of a powerful cupping glass to a recently poisoned wound, would prevent the absorption of the poisonous matter."

In this second Essay on Absorption, Dr. Barry has presented us with a short but entertaining account of the historical evidences upon the utility of the application of cupping glasses to poisoned wounds; a practice which, although very ancient, appears to have been employed without any correct theoretical notions of the mode in which the benefits acknowledged to be derived from it were produced.

---

*An Elementary System of Physiology.* By John Bostock, M.D. P.G.S. F.R.S. L.S. and H.S. M.R.I.; Mem. and late V. Pres. of the Medical and Chirurgical, and Mem. of the Astronomical and Meteorological Societies of London; Mem. and late Pres. of the Edinburgh Medical Society; Hon. Mem. of the Literary and Philosophical, and of the Historical Societies of New York; Lecturer in Chemistry at Guy's Hospital; Professor of Physiology in the Liverpool Royal Institution, &c. &c. Vol. II.—8vo. pp. 643. London: Baldwin, Cradock, and Joy. 1826.

The last few years have been very prolific in physiological writings: we have had translations of Blumenbach and Rudolphi, of Richerand and Magendie: the work of Dr. Bostock, however, still remains the only one which is *indigenous.* The second volume of this has just appeared, after rather an alarming interval: this we are informed arose from unavoidable circumstances, and we are led to hope that the third volume will succeed the present without any considerable delay. The plan of publishing a work of this kind in successive parts, is liable to many objections: few choose to purchase a single volume while others are to come, and thus many, who would be glad to avail themselves of it, postpone doing so till the system be completed. If a considerable interval be suffered to elapse, the doctrines contained in the first part may have become obsolete before the continuation sees the light. We would, therefore, respectfully suggest to the author, in justice to himself and to his readers, the propriety of completing his system as speedily as is consistent with the labour and difficulty of the task.

The volume before us, like the one which preceded it, manifests great industry and research, joined to fair and judicious criticism. It is in many respects essentially different from, and superior to, the works of the continental physiologists, to the translations of which we have above alluded. In these, each writer is guided by his own particular views, which gives to their works more of individuality than ought to appear in a general system: thus, in Rudolphi we have too much of the *organismus,* and in Magendie the little word *moi* becomes rather an eye-sore. This is not the case in the work of Dr. Bostock, in which is contained a very elaborate account of the leading doctrines in physiology, with very copious references to the writings of different authors; so that the student will derive from its perusal a much more general knowledge than from any other work in the English language.

The subjects discussed in the present volume are *Respiration, Animal Temperature, Secretion, Digestion,* and *Absorption.*

---

*Modern Domestic Medicine; or, a Popular Treatise illustrating the Character, Symptoms, Causes, Distinction, and correct Treatment, of all Diseases incident to the Human Frame; embracing all the modern Improvements in Medicine, with the Opinions and Practice of the most distinguished Physicians. The whole intended as a Medical Guide for the use of Clergymen, Families, and Students in Medicine. To which is added, a Domestic Materia Medica; a Description of the Virtues, and correct Manner of using the different Mineral Waters of Europe, and the Cold, Warm, and Vapour Baths; a copious Collection of approved Prescriptions, adapted to domestic use; and a Table of the Doses of Medicines.* By Thomas John Graham, m.d. Member of the Royal College of Surgeons, &c.—8vo. pp. 551. London: Simpkin and Marshall. 1826.

Of the numerous shapes which puffing and quackery assume, Treatises on "Domestic Medicine" are the most injurious to the public: these books profess to give what never can be obtained, but as the result of long and laborious study in the closet, and observation at the bedside of the patient, "a clear and correct description of the nature, symptoms, causes, distinction, and most approved treatment, of the diseases to which the human frame is liable." Every man, at all acquainted with his profession, must be aware that there is no "royal road" to practical knowledge, and that all such attempts are absurd and mischievous. But, if the public are losers by the imperfect and inaccurate information they thus obtain, the medical profession at least ought to be grateful to the manufacturers of popular Treatises, for the increase of business which they procure them. A patient who has long tampered with his constitution,—who has given a complaint, originally slight, time and opportunity to become severe, or an acute disease to pass into a chronic form,—such patient, we say, is greatly more lucrative in the end than one who applies to his medical attendant on the first symptoms of indisposition.

With regard to the work more immediately before us, it is like all the others on the same subject—a compilation of short popular descriptions of disease, with similar brief and superficial accounts of the treatment. Turning to Cholera (the disease of the present season), we were rather astonished to find, as one of the methods of treating it, that "*forty, sixty, or eighty drops of the diluted acid (sulphuric) may be given in water every two or three hours.*" Upon the whole, however, it is not inferior to its predecessors in the same line; so that, if any unprofessional person will be so foolish as to perplex himself with medical studies, he is likely to do as little mischief with the work before us, as by taking any other for his guide. At the same time, we would just mention, in conclusion, that FALRET, in his excellent Treatise on Hypochondriasis and Suicide, has enumerated among their exciting causes, "Lecture habituel de Buchan," (BUCHAN's Domestic Medicine;) and, of course, the observation applies equally to all others. Very good

### COLLECTANEA.

Floriferus, ut apes, in saltibus omnia libant,
Omnia nos, itidem, depascimur aurea dicta.

